# Supplementary material for: Sex and HDAC4 Differently Affect the Pathophysiology of Amyotrophic Lateral Sclerosis in SOD1-G93A Mice
Source: Int J Mol Sci. 2022 Dec 21;24(1):98. doi: 10.3390/ijms24010098 (PMC9820722; doi:10.3390/ijms24010098)
Supplement: Supplementary file 1 [file ijms-24-00098-s001.zip › ijms-2064245-supplementary.pdf]

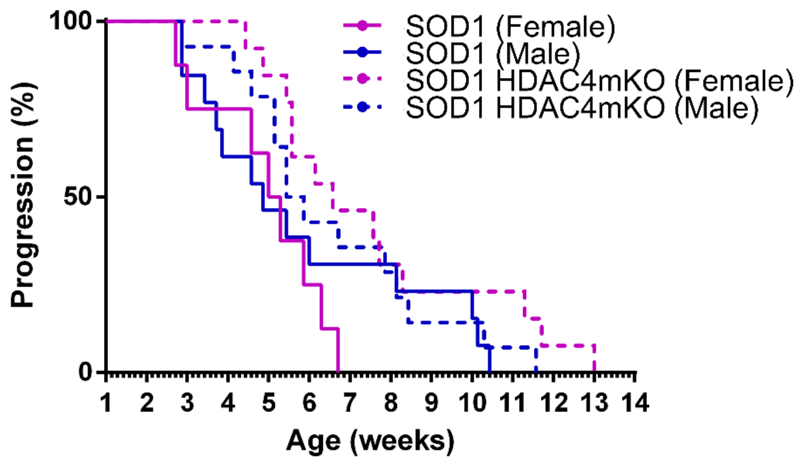

Figure S1. Neither sex nor HDAC4 affects ALS progression in SOD1 mice. ALS progression of SOD1 and SOD1 HDAC4mKO mice. n=14 males for each genotype; n=12 females for each genotype.
